# Simulated case management of home telemonitoring to assess the impact of different alert algorithms on work-load and clinical decisions

**DOI:** 10.1186/s12911-016-0398-9

**Published:** 2017-01-17

**Authors:** Illapha Cuba Gyllensten, Amanda Crundall-Goode, Ronald M. Aarts, Kevin M. Goode

**Affiliations:** 1 Personal Health Solutions, Philips Research, p.030, High Tech Campus 34, Eindhoven, 5656AE Netherlands; 2 Signal Processing Systems, Eindhoven University of Technology, Eindhoven, The Netherlands; 3 Dept. of Nursing, Faculty of Health & Social Care, University of Hull, Kingston-Upon-Hull, UK; 4 Dept. of Health Professional Studies, Faculty of Health & Social Care, University of Hull, Kingston-Upon-Hull, UK

## Abstract

**Background:**

Home telemonitoring (HTM) of chronic heart failure (HF) promises to improve care by timely indications when a patient’s condition is worsening. Simple rules of sudden weight change have been demonstrated to generate many alerts with poor sensitivity. Trend alert algorithms and bio-impedance (a more sensitive marker of fluid change), should produce fewer false alerts and reduce workload. However, comparisons between such approaches on the decisions made and the time spent reviewing alerts has not been studied.

**Methods:**

Using HTM data from an observational trial of 91 HF patients, a simulated telemonitoring station was created and used to present virtual caseloads to clinicians experienced with HF HTM systems. Clinicians were randomised to either a simple (i.e. an increase of 2 kg in the past 3 days) or advanced alert method (either a moving average weight algorithm or bio-impedance cumulative sum algorithm).

**Results:**

In total 16 clinicians reviewed the caseloads, 8 randomised to a simple alert method and 8 to the advanced alert methods. Total time to review the caseloads was lower in the advanced arms than the simple arm (80 ± 42 vs. 149 ± 82 min) but agreements on actions between clinicians were low (Fleiss kappa 0.33 and 0.31) and despite having high sensitivity many alerts in the bio-impedance arm were not considered to need further action.

**Conclusion:**

Advanced alerting algorithms with higher specificity are likely to reduce the time spent by clinicians and increase the percentage of time spent on changes rated as most meaningful. Work is needed to present bio-impedance alerts in a manner which is intuitive for clinicians.

**Electronic supplementary material:**

The online version of this article (doi:10.1186/s12911-016-0398-9) contains supplementary material, which is available to authorized users.

## Background

Patients with chronic heart failure (HF) require careful clinical management in order to reduce the impact of their symptoms, avoid unplanned hospitalisations and improve their survival [1]. Data from the ESC-HF Pilot study [2] has shown that compared to outpatients with HF, those discharged from hospital after an episode of acute HF, are almost two and a half times more likely to die within one year and almost twice as likely to be re-admitted due to exacerbation of HF. Home telemonitoring (HTM), set within a system of integrated care, offers a strategy to manage medium-to-high risk patients with HF. Current HTM systems for HF management enable the daily measurement of weight, blood pressure and heart rate which is then fed back to a healthcare professional to assess whether changes to the patient’s care is required.

Most meta-analyses of randomised control trials (RCT) of HTM for HF [3–6] have concluded that compared to usual care, HTM reduces all-cause and HF-related hospitalizations and reduces all-cause mortality. However, there was significant heterogeneity between the RCTs included in these analyses [7], with many showing no benefit over usual care; a similar conclusion found in recent larger trials [8–10]. The differences in outcomes between trials are likely to be multifactorial and may be attributable to inappropriate choice of HTM service model, inappropriate patient selection, lack of multi-disciplinary team integration and co-ordination and inadequate alert management [11].

In HTM systems, an alert is generated when daily measurements fall outside of pre-specified limits for a given patient. These limits (or parameters) are usually specified by the healthcare professional in charge of patient care – typically the general practitioner, community matron or HF nurse specialist. If the limits are set too wide, then there is a high risk that a patient will have decompensated before an alert is generated; set them too tightly and this will lead to many false alerts. Since setting limits too wide could compromise patient care, it is understandable why conservative limits might be chosen. However, a recent study found that up to 97% of the alerts generated in their HTM system were not attributable to cardiac related changes in medication or hospitalisation [12]. This may have two unforeseen consequences; alert complacency and increased workload for the healthcare professional monitoring the HTM data.

If the system keeps saying there is a problem when there is none, the HTM healthcare professional becomes desensitised to the alerts and then may miss a problem when it does arise. Obviously if the system is generating many more alerts than need acting upon, this greatly increases the work load of the monitoring staff. This will limit the size of the patient case-load that can be managed or result in missed “true” alerts, due to the volume of alerts that have to be reviewed and processed. The net consequence of these problems is that patient outcomes will be compromised, some hospitalisations will not be avoided and the costs of implementing HTM will spiral as the staff required to manage the ever increasing volume of alerts has to grow.

Daily weight monitoring is recommended for patients with HF [1] and there are various published rule-of-thumb guidelines for clinically important weight gain that may indicate decompensated HF (summarised by Goode et al. [13]). However, recent evidence demonstrates that data-driven approaches looking at trends and patterns of weight change, improve the accuracy of detecting patient deterioration when compared to these simple expert rules [13–16].

Weight itself may not be the best measure of fluid congestion and the detection of decompensated HF can be improved by using non-invasive trans-thoracic impedance [16] implanted intra-thoracic impedance [17] and haemodynamic pressures [18, 19]. However, whatever system is used to monitor the patients, ultimately it is the care decisions made by the patients and the healthcare professionals which improve clinical outcomes. Despite this, there has been surprisingly little research focused on the actions of the healthcare professional on how they review the telemonitoring data they receive [20].

We designed the present study to compare the time and actions taken by telehealth clinicians to review patients using different methods of alerting in a simulated setting. Simulation methodologies for estimating HTM workload have been presented [21], but no study has looked at the simulation of HTM data to investigate the actions of the healthcare professionals in response to suspected decompensation alerts. In this study we used previously collected data from an observational telemonitoring trial to construct a simulated monitoring workstation. This was used to compare the time a telemonitoring clinician spent reviewing HTM data and what clinical actions were recommended between different alert strategies. Our hypothesis was that improved detection algorithms and novel sensor modalities would improve the workload, through reduced time spent reviewing cases and calls to patients, thus increasing the focus on patients which are genuinely deteriorating.

## Methods

### Data source for case-load simulation

The data used to generate the simulated case-load was collected at six HF-clinics in Germany and Spain as part of the MyHeart heart failure management observational study [22] Patients were included in this study if they had chronic HF with elevated levels of the N-terminal prohormone of the brain natriuretic peptide (NT-proBNP ≥ 500 pg/ml), were taking at least 40 mg/day of furosemide or equivalent and were in New York Heart Association (NYHA) functional class II, III or IV.

Participating patients were required to; (a) answer two daily symptom questionnaires using a personal digital assistant, once in the morning and once in the evening and (b) take daily measurements of weight, blood pressure and trans-thoracic impedance (TTI) using a wearable vest. The study was purely observational and no treatment decisions were made based on the data logged. In total, 91 of the patients enrolled in the MyHeart study had sufficient compliance with the measurement system for their data to be included in this simulation; of which 70% were men with a mean (±standard deviation) age of 63 (±12) years, body mass index of 29 (±6) kg/m^2^ and left ventricular ejection fraction of 31 (±12) %. On average these patients were monitored for 10 months.

### Thoracic fluid information from monitoring of trans-thoracic impedance

TTI measurements are sensitive to the amount of fluids in the tissue as fluids increases the conductivity of the tissue [23, 24]. However, uptake of this technology has been slow due to cumbersome prototype technologies and difficulties in interpreting the results since electrical resistance does not directly translate into a lung water assessment [25]. Recent analysis of the MyHeart trial data suggests that non-invasive TTI is more sensitive to impending deterioration than standard measures of fluid accumulation such as weight [16].

### Different methods of deterioration detection: alert algorithms used in simulation

Three different alert algorithms to prompt patient reviews was used in this simulation experiment; a rule-of thumb algorithm using weight (*weight-RoT*) based on the ESC guideline, and two advanced algorithms: a trend algorithm using weight (*weight-MACD*) and a trend algorithm using trans-thoracic impedance (*impedance-CUSUM*). These are described in more detail below. Whilst the weight-MACD and impedance-CUSUM have been shown to be more effective in the detection of impending deterioration [13, 16], they may not be as easy to interpret as simple differences, which could paradoxically result in longer review times and worse decisions.

#### Weight-RoT

Monitoring weight changes caused by fluid retention is routinely recommended for HF management and different algorithms for alerting caregivers to potential worsening HF have been suggested [1, 13, 26]. Typically, the difference between the current and past weight measurements is used to decide whether the patient needs reviewing and/or whether changes to their management made. An increase of 2 kg or more in the past 3 days was used for this algorithm, as recommended in the ESC guidelines [1].

#### Weight-MACD

Trend detection algorithms have been suggested to improve detection of fluid retention by removing much of the inherent variability found in difference measures and instead look at the trend of change over longer time periods [13, 14]. For this study, the moving average convergence divergence (MACD) trend algorithm was used. This algorithm looks at the differences between two exponentially weighed averages with different time-horizons; one over a long-period and one over a short one. The use of such an algorithm has been shown to improve specificity in detecting worsening HF [13]. The parameter settings for the MACD algorithm were chosen in order to optimise the sensitivity and specificity of detecting impending hospitalisation for worsening HF. The alert threshold was set to be 0.54, which is slightly lower than that defined previously using the MyHeart data [16], and will therefore generate slightly more alerts.

#### Impedance-CUSUM

Another trend-detection method from process control is to use cumulative sums to detect changes. For this study, the cumulative sum control chart (CUSUM) was used which compares the deviation from a moving average normalised for standard deviation to establish whether a change has occurred [27]. This method has been successfully applied to intra-thoracic impedance and pulmonary pressures [27, 28]. Again the parameter settings for the CUSUM algorithm were optimised, with the alert threshold set to −7, slightly lower than reported previously using the MyHeart dataset [16].

### Data preparation

**Fig. 1:**
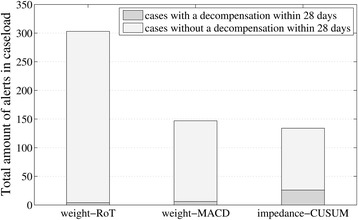
The total amount of alerts generated for the monitored patients divided into alerts in which patients decompensated within 28 days and those in which patients did not

The three algorithms described above were applied to the MyHeart data to create alerts indicating possible deterioration of heart failure. For each of the algorithms, the measurement data was segmented into 28-day episodes that ended in an alert. This process generated 556, 314 and 287 episodes using the weight-RoT, weight-MACD and impedance-CUSUM algorithms respectively. In patients with alerts occurring on consecutive days, we removed every 2nd and 3rd episode to minimise the chances of showing the same alert period within a given case-load simulation. This procedure generated 303, 147 and 134 episodes to be reviewed in the weight-RoT, weight-MACD and impedance-CUSUM arms respectively, these together with the alerts belonging to cases decompensated are presented in Fig. 1.

The resulting alert episodes were divided randomly into 15 groups (or case-load snapshots) and each episode assigned randomly to a fictitious patient name. A proportional amount of episodes containing no alerts within a 28-day windows, were selected from the remaining data and combined with the alert episodes to create 15 virtual case-loads of patients in each of the study arms. The patient episodes without alerts serve no real function within the study other than to create the impression of a real case-load; some patients having alerts and some not.

### Simulated telemonitoring station

**Fig. 2:**
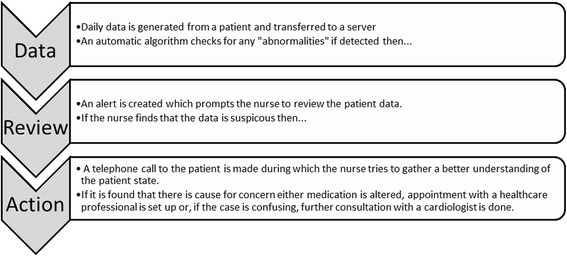
Telemonitoring process assumed for the study

The simulated HTM data and resultant alerts was presented to the participants using a simple interactive graphical user interface (GUI) that has the feel of a real HTM system. The resultant GUI was designed to be simple yet understandable and capture the telemonitoring process steps given in Fig. 2. The final GUI design was arrived at iteratively. Firstly, a non-functional GUI prototype based loosely on the system used by HTM nurses at Castle Hill Hospital (UK) was shown to a HTM nurse for review and comment.

**Fig. 3:**
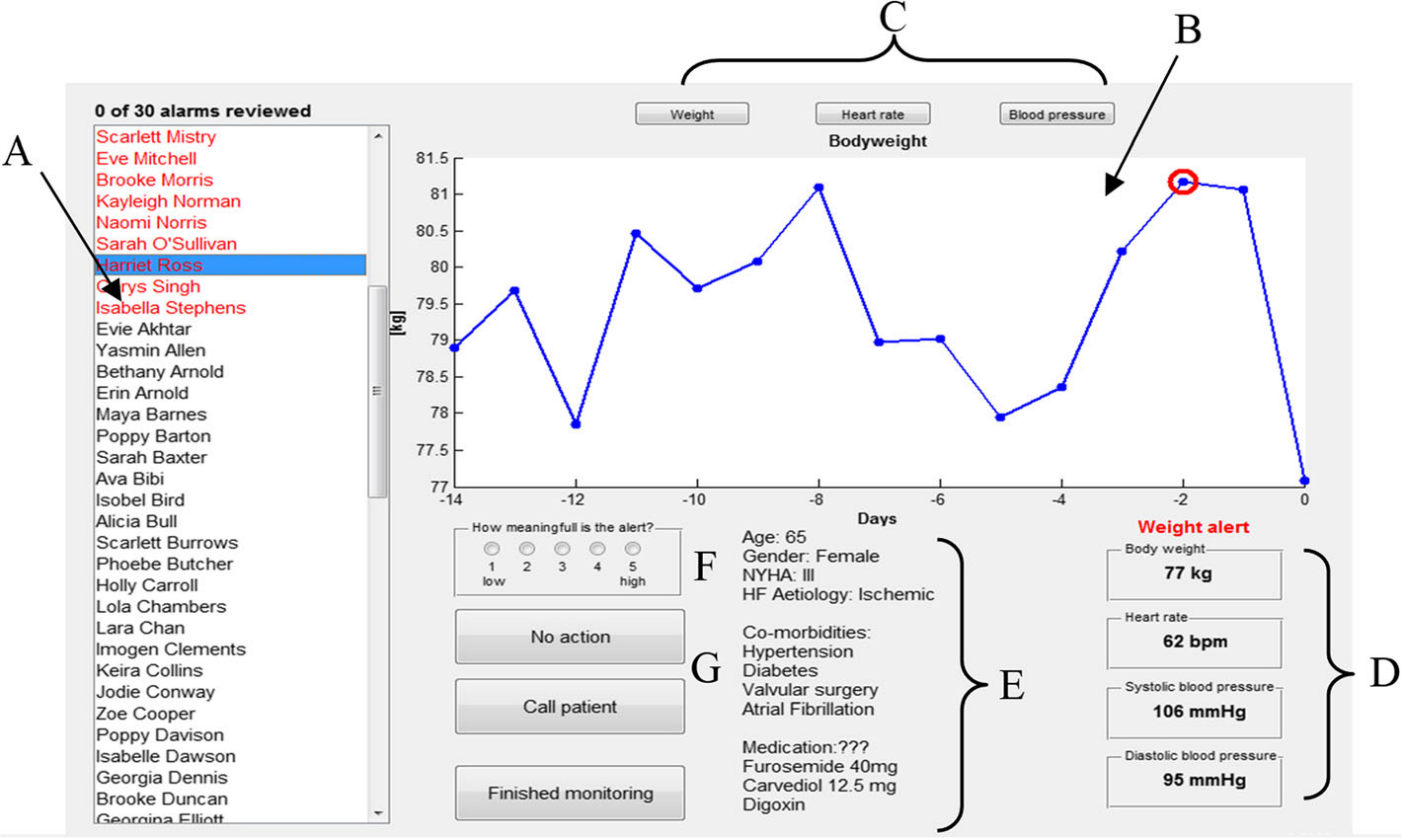
Final GUI design – all names displayed are fictitious. Key: **a**. List of patients in case-load. Patients highlighted in red have alerts that need to be reviewed. **b**. Measurement data review panel. Data points circled in red indicate an alert. **c**. Buttons to review other measurement data. This will appear in panel B. **d**. Most recent HTM measurements. **e**. Patient demography, co-morbidities and current medication. **f**. Participant rates the meaningfulness of the alert here. **g**. Response buttons: No Action or Call Patient

Recommendations from this nurse were then incorporated into the next design iteration and the GUI was loaded with some test data. This was then shown to a second nurse (ACG) for further review and comment on the functionality of the GUI design. The final GUI design, after incorporating the suggestions of both nurses, with the addition of some additional adjustments, is shown in Fig. 3.

During a run of the simulation, the case-load of patients is displayed in the panel to the left (labelled A on Fig. 3). Those patients that have generated an alert are highlighted in red at the top of this list and will need to be reviewed by the HTM nurse/clinician. When a patient is selected the daily measurement data will be displayed in the main panel (B). For participants randomised to either of the weight algorithm arms, this will display weight data for the preceding 28 days by default; with red circles indicating alerts that have been raised in the past three days. Similarly, if they are randomised to the trans-thoracic impedance arm, they will see the trans-thoracic impedance data by default. By clicking on the buttons (C) above panel B, the nurse/clinician can review the other daily measurement data (i.e. blood pressure, heart rate and weight). In the weight algorithm arms, the trans-thoracic impedance data remains hidden. The values of the most recent HTM measurements (D) are shown below panel B on the right-hand-side. To the left of this (E), basic patient information is provided such as age, sex, NYHA class, HF aetiology and co-morbidities.

Once the data have been reviewed by the nurse/clinician they can then rate how important they felt the alert was using a five point Likert scale (i.e. l = low to 5 = high) (F). They then indicate what action they would take in response to the alert. If they considered the alert to be of no concern, they can click *No Action* and then move on to the next patient. If they do have concern, they can opt to *Call Patient* (G).

#### Simulated call

**Fig. 4:**
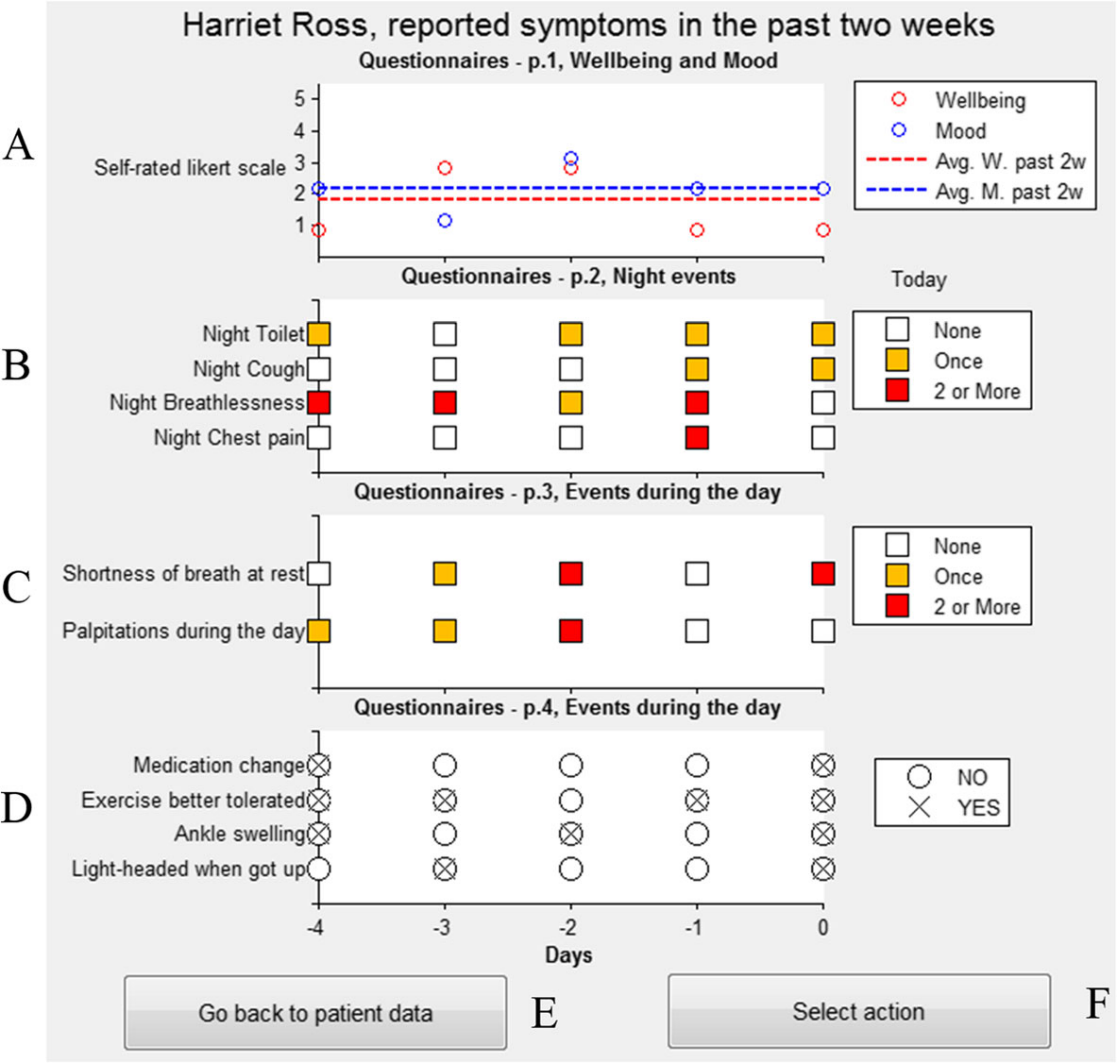
Simulated call report of past symptoms and self-reported health and wellbeing. Key: **a**. Self reported wellbeing and mood in the past 5 days. **b**. Multiple choice questions covering night symptoms in the past 5 days. **c**. Multiple choice questions covering day time symptoms in the past 5 days. **d**. Day time symptoms/medication change with a YES/NO answer in the past 5 days. **e**. Takes the study participant back to the alert review window. **f**. Select the decision of what to do with this patient

Since the data used in this simulation was retrospective, no actual calls can be made. To emulate the type of information a call might generate, the data from the comprehensive symptom questionnaires collected during the MyHeart trial was used to create an overview of a patient’s symptoms during the previous 5 days, see Fig. 4. The patients in MyHeart were asked to rate their general mood (*How was your mood today?*) and wellbeing (*How was your general well-being today?*) on a 5-point Likert-scale. Responses for the past 5 days were combined with reference lines for the average mood and wellbeing over the preceding two weeks (labelled A on Fig. 4). Multiple choice questions covering symptom levels (*Did you have to go to the toilet? (at night); Did you have any coughing last night?; Did you experience breathlessness last night?; Did you experience any chest pain? (at night); Did you feel shortness of breath at rest today?; Did you experience palpitations today?*) are color-coded according to their frequency i.e. none = white, once = yellow, twice or more = red (labelled B and C). Questions having only a single answer (*Do you feel more ankle or leg swelling than yesterday?; Have you changed your medication?; Did you tolerate exercise better today than yesterday?, Did you feel light-headedness when getting up this morning?*) were marked with a cross for yes or empty circles for no (labelled D).

#### Logging of decision

Having “called” the patient, the nurse/clinician can either click *Go back to patient data* (E) to further review the monitoring data or click *Select action* (F) and indicate what action they recommend from the following options;

1. High level of concern, e.g. send a community nurse to the patient
2. Raised level of concern, e.g. increase the dosage of diuretics
3. Beginning level of concern, e.g. close follow-up in the following days
4. Low level of concern: no further action.

However, if the participant feels that these suggested responses do not adequately capture the preferred action they had an option to comment further.

The time spent reviewing each patient is logged automatically by the simulator. Once a case-load of alerts has been reviewed the participant can click on *Finished monitoring* and the next case-load will be presented or they have the option to pause the simulation. The complete experiment for each participant was expected to last 2–3 h.

### Study design

We recruited healthcare professionals (nurses and doctors) in the United Kingdom with experience of managing patients with heart failure using HTM. Participants were recruited via nursing, heart failure and telehealth networks known to the authors, including Twitter, Linked-In and other social media sites.

Potential participants were asked to complete an online questionnaire (using Survey Monkey) to establish their level of experience of managing patients with HF and use of HTM systems. Participants with experience of both HF management and HTM were then randomised to receive alerts generated using one of the described algorithms. They were sent a self-extracting application package containing the simulation program together with instructions on how to install it, how to conduct the experiment and their randomization code. Participants randomised to the trans-thoracic impedance arm also received a short educational video to explain the measurement technology and how to interpret the impendence signal, since only a few participants would’ve been familiar with this measurement modality.

Each participant was then presented with 15 case-loads to review using the simulation system. The order in which the cases were presented to the participants was identical within each study arm.

### Statistical considerations: study size, simulation time and analysis

This was a pilot study and with the difference in amount of alerts between the trend algorithms and the rule-of-thumb method we anticipated that only a small number, eight participants in each arm, would be adequate to show differences (see Additional file 1: Appendix 1 for the statistical argument), therefore we aimed to recruit approximately 10 participants in each arm.

Participants were randomised following a randomised string in the order they agreed to take part in the study with dropouts after agreement appended at the end of the string. The primary hypothesis was that the reduction in alerts by applying a trend algorithm or a novel marker will lower the total time spent in the simulated interface (i.e. weight and impedance trend alerts vs. standard alerts). A Mann–Whitney *U*-test was used with a significance level of 0.05. Secondary analysis included: the suggested interventions (e.g. call GP, review medication, etc.) and the rated importance of the alerts. Since not all participants are monitored during the experiment they might leave the simulation, e.g. to get a cup of tea or coffee, without pausing. This would result in excessively long review times for specific alerts. We therefore limited the maximum time to review an alert to five minutes, thus any review time lasting longer was marked as lasting five minutes. Similarly, sometimes participants might miss to review an alert which would lower their total review time. In these cases we imputed the average review time for that alert.

## Results

### Participants

During the period from the 13th of May 2013 to the 24th of March 2014 a total of 99 potential participants were contacted or responded to recruitment ads to participate; of these 35 declined participation; four were found to have low telehealth experience and excluded and 27 did not respond. The remaining 33 agreed to take part and were schedule for a visit or got sent the simulation software, according to preference. A further 9 declined after receiving a package and seven dropped out. In total we received complete data from 16 clinicians (8 using *weight-RoT* (arm A), 4 using *weight-MACD* and 4 using *impedance-CUSUM* (together arm P)) a corrupted file was also received. With respect to the low response rate we decided to halt recruitment and analyse the resulting data by grouping the two advanced arms together. In the pre-specified analysis plan a sample of 8 was deemed sufficient for the primary analysis, which was reached by pooling. Therefore it was decided that, at least for the primary analysis of total review time, the analysis would still be feasible. Furthermore, many usability issues can already be detected with five participants [29]. Participants were mostly specialised Heart Failure nurses with high self-rated experience. Summary characteristics are presented in Table 1.

**Table 1:** Characteristics of the recruited participants. M indicates those receiving the advanced weight alert (*weight-MACD*) and C those receiving the advanced impedance alert (*impedance-CUSUM*)

	Arm A	Pooled Arm P
Number	8	8 (4 + 4)
Self-entered occupation	1 Nurse lecturer	1 Senior research nurse (M)
	1 Cardiac specialist nurse	1 Heart failure specialist nurse (M)
	2 Heart failure nurse specialist	1 Telehealth nurse (research) (C)
	1 Cardiac nurse specialist	1 Heart failure nurse (M)
	1 Heart failure nurse	1 Clinical lecturer telehealth (C)
	1 Research nurse	2 Heart failure nurse (C)
	1 Did not answer	1 Trust doctor (cardiology) (M)
How would you grade your knowledge of telemonitoring systems?	4 Expert	4 Expert
	2 Intermediate	3 Intermediate
	2 Did not answer	1 Novice
How would you grade your knowledge of heart failure treatment and pathophysiology?	4 Expert	6 Expert
	2 Intermediate	2 Intermediate
	2 Did not answer	

### Time spent reviewing alerts

The total time spent reviewing the data for the simple alerts (arm A) was 149 ± 82 min (mean and standard deviation) which was higher than for the advanced alerts (arm P) 80 ± 42 min (p = .038) due to the larger amount of alerts in arm A. The time spent per alert for the pooled arm P compared to arm A was: arm A, 29.5 ± 16.3 s; arm P, 34.1 ± 17.0 s, p = .505; implying that the review of advanced alerts takes a similar amount of time as simpler alerts. However, there was substantial variability between participants (see Additional file 3: Appendix 3). We observed a training effect, where the first caseload took longer to complete than the following caseloads, but this did not influence the result. We tested this by replacing the first caseload review times with the average time in subsequent caseloads for a given participant; resulting in lowered review times but no difference for the statistical tests (total time: 135 ± 82 min vs. 70 ± 32, *p* = .038; per alert time: 26.8 ± 16.3 s vs. 30.1 ± 13.6 s, *p* = .328).

### Rated importance of alerts, recommended actions and agreement between participants

**Fig. 5:**
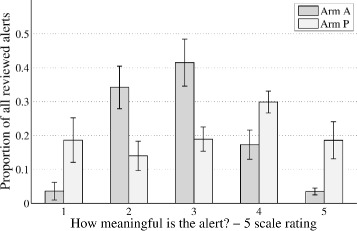
The mean and standard error of the proportion of the different ratings suggested for the reviewed cases by the clinicians in each arm

The distribution of rating scores given to the alerts in the two arms differed, see Fig. 5. In general, the ratings from the pooled arm P was much more uniform, with reduced medium alert ratings (2 and 3), increased high ratings (5), but also increased amount of very low ratings (1). The proportion of alerts given a specific rating by the clinicians in arm A vs arm P was (mean and standard deviation): rating 1, 3.6 ± 7.3% vs. 18.6 ± 18.5%, *p* = .027; rating 2, 34.2 ± 17.8% vs. 14.0 ± 12.3%, *p* = .010; rating 3, 41.5 ± 19.6% vs. 18.9 ± 10.1%, *p* = .007; rating 4, 17.3 ± 12.1% vs. 29.9 ± 9.2%, *p* = .065; rating 5, 3.5 ± 2.8% vs. 18.6 ± 15.5%, *p* < 0.001. If the rating scores were averaged for each clinician then differences in mean rating were slight (Arm A 2.83 ± 0.30 vs. Arm P 3.16 ± 0.69, *p* = .442).

The proportion of actions where the clinician indicated concern i.e. suggested that the alert needed further follow-up, medication change or send a community nurse, are presented in Table 2 together with raw-agreement (i.e. the percentage of possible rater-pairs agreeing). In general only a slight majority of the possible rater-pairs agreed on whether further follow-up was needed or not, and the kappa values were around 0.3 which is usually considered poor agreement.

**Table 2:** Proportion of alerts (mean and standard error) given either a suggestion of no action (i.e. no concern indicated either directly following review of the data or after the simulated call) or action (concern indicated with either follow-up, medication adjustment or sending a community nurse) together with the percentage of raw agreement (i.e. the average proportion of rater-pairs agreeing of the total possible rater-pair agreements) with bootstrapped 95% confidence intervals. The Fleiss kappa statistic excluded cases in which not all clinicians provided a rating (total: arm A, 281 cases with 8 raters; arm P, 147 + 85 cases with 4 + 4 raters)

Indicated response		Arm A	Arm P
No action	Proportion of alerts	50.5 ± 6.2%	40.6 ± 7.1%
	Agreement across participants on response	66.8% (63.4–69.8)	57.5% (52.1–62.4)
Action (concern indicated)	Proportion of alerts	49.5 ± 6.2%	59.4 ± 7.1%
	Agreement across participants on response	66.4% (62.7–69.6)	72.8% (69.0–76.3)
	Fleiss’s Kappa statistic	.334 (.329–.340)	.305 (.291–.319)

**Fig. 6:**
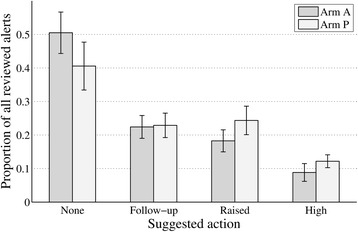
The mean and standard error of the proportion of the different actions suggested for the reviewed cases by the clinicians in each arm

The proportion of suggested actions by category between arm P and A is presented in Fig. 6. No large differences between the two arms could be detected. However, when the individual algorithms were differentiated in the advanced arm it became clear that participants randomised to the advanced bio-impedance alerts seldom indicated actions in contrast to the advanced weight alert where many alerts got an action (Additional file 2: Appendix 2: Figure 7).

In the detailed agreement analysis were the advanced arm was split into the two algorithms and each action considered its own category (Additional file 2: Appendix 2: Table 3) the agreements were highest for the “No Action” group in arm A and those having the advanced bio-impedance alert, suggesting that the clinicians agreed that these alerts were of little concern.

Although based on a limited amount of participants, the finding of few suggested actions and high agreement on alerts of no-concern in the bio-impedance arm is concerning. It is known that the rule-of-thumb approach to weight (Arm A) is quite insensitive to deterioration and this might be recognised by the clinicians who sort out these cases, which can explain the low ratings and actions in arm A. However, Impedance is a much more sensitive marker of decompensation, and many of the reviewed cases do in fact deteriorate, but this did not seem to be recognised.

### Actions suggested for alerts which subsequently led to decompensations

It is important that alerts in unstable patients, who subsequently got hospitalised, are adequately responded to. The mean significance rating given to the alerts for patients who later decompensated was higher compared to those patients that did not decompensate (difference 0.36 ± 0.45, *p* = .013). However, when split by allocation arm, this difference was only detected in arm P (difference 0.66 ± 0.18, *p* = .008) and not in arm A (difference 0.06, ± 0.44 *p* = .64). More than half the alerts given to patients who later decompensated got an action indicating raised or high concern (53.3%) compared to slightly less than a third in the more stable group (28.7%). Within the pooled arm this difference was most pronounced in those randomized to the advanced weight algorithm were 79.2% of the alerts in patients who subsequently decompensated got an action indicating raised or high levels of concern, compared to 47.0% for the advanced impedance algorithm.

## Discussion

The field of HTM for heart failure management has seen increased focus in the past decade with several large RCTs testing its efficacy. However, the benefits of HTM still remains a topic of controversy [30]. Alert strategies commonly differ between trials and there is little evidence supporting a threshold of, for example, 2 kg in 3 days. Several studies have suggested that algorithms using weight can be improved by more advanced processing [13, 14, 16] and that modalities such as bio-impedance could improve this further. However, the question still remains whether such alerts would be as interpretable and lead to better care decisions. We conducted this pilot trial to explore benefits of advanced alert algorithms as compared to the more commonly used simple alert algorithms in time spent by clinicians and their suggested actions. In a simulated setting, we found that more advanced alerts did lead to a reduced time spent reviewing, approximately halving the total review time, and a higher proportion of alerts receiving the highest rating by the clinicians.

However, agreement between clinicians on whether an alert required further action was only moderate, with 60–70% of rater-pairs agreeing resulting in a Fleiss’s kappa value around 0.3. The distribution of ratings varied between the two groups, with the more advanced algorithms having a much more uniform distribution of ratings from 1, low, to 5, high, compared to the simple alert setting were most alerts where considered to be of low or moderate meaningfulness (2 and 3). Therefore, although the advanced arm had a larger proportion of alerts with high ratings of 4 and 5 there were also more alerts that received the lowest rating. In particular, participants randomised to the bio-impedance type of advanced alerts considered close to half of them to not be of particular importance, despite the algorithms showing high sensitivity in previous studies. This is highlighted by a back-of-the-envelope calculation of the proportion of decompensation that would receive an action of high or raised concern. We calculated this by multiplying the sensitivity of each algorithm to alert of an upcoming decompensation (13% for rule-of-thumb, 33% for the MACD weight algorithm, 60% for the impedance CUSUM algorithm) [16] with the proportion of suggested actions of raised or high concern for these cases. For the respective algorithms this percentage was: weight rule-of-thumb, 6%; MACD weight, 25%; bio-impedance CUSUM, 27%. Thus, despite its high sensitivity, the bio-impedance alerts performance was similar to the weight-MACD algorithm. Although the absolute values of these estimates are associated with considerable uncertainty the relative comparison of their magnitudes demonstrates the important interplay between algorithm sensitivity and the response by the clinician. The data and the alert needs to be designed in a manner which supports the decision making process of the clinician. Perhaps the lack of benefit in recent studies using advanced alerts from impedance measurements done with implanted cardiac devices [31, 32] might, in part, be attributed to the difficulty of interpreting the output measure and guide intervention.

We conclude that advanced alert algorithms are a promising avenue to improve telemonitoring systems but bio-impedance alerts may need further refinement. A focus on alert presentation and handling could be one of the factors that need optimization for bio-impedance measures. Alerts based on thoracic bio-impedance may precede clear symptoms [33] and it possible that this was not appreciated by the participants. Perhaps using the trends alerts together with guide values of dry and wet impedance [34, 35] values would improve the clinicians’ ability to interpret the changes and contextualise it with symptoms and other signs of deterioration. Furthermore, the moderate agreement between clinicians suggest that weights are given to different aspects of the presented cases, at least in this simulated program. This finding needs further confirmation in a setting closer to a real-world implementation of a HTM service so that the reasons for a lack of homogeneity and quality of telemonitoring alert handling can be studied in more detail. A suggestion could be to implement retrospective reviews within a group of telehealth nurses to evaluate and discuss actions and outcomes of prior alerts.

### Study limitations

This is a pilot study and therefore only exploratory in nature. The study intended to reach a larger sample size (30) then what was ultimately achieved. Therefore the advanced alerts needed to be pooled to compare them to the simpler alert and differences between the two advanced algorithms have small samples. There was a larger drop-out in the advanced arms, whereas the randomization process would have expected roughly equal response rates. It is possible that some users were less able to interpret the output of the advanced alerts data, and dropped out because of this. Which could be interpreted to further support the finding that some alerts where not regarded as useful, or difficult to interpret in the advanced arms.

During the simulation run all actions were logged and reviewed however the participants were not observed in person by an investigator and thus we could not control for possible distractions. It is possible that this could have caused a lowered agreement between participants. Furthermore, the participants were not always known to the investigators and the remote participation also only allowed for identity confirmation through mailed letters of signed consent.

By being a simulation study it, by definition, foregoes accuracy to be able to re-test and tune scenarios which might not always be possible in the real-world. For example, the GUI lacks some of the functionality that you might expect to find on commercially available HTM workstations and the nature of a simulation is that certain responses you might take in real-life cannot be emulated easily.

For patients randomised into the bio-impedance arm no alerts associated with weight measurements was shown, although the weight data itself could be reviewed by the participant. In real practice it might be advantageous to provide alerts in other vital signs that provide corroborating evidence of deterioration. However, in an effort to simplify the analysis to reflect the impact of the bio-impedance alerts only, the weight alerts were omitted.

The study participants were not be able to call a patient to discuss the reasons behind an alert. Whilst we have attempted to emulate the type of information that such a call might elicit, the time spent on a real call may be very different. Patients may often talk longer in a real call about issues unrelated to HTM that reflect emotional and social aspects of their care. Therefore one could argue that participants might have a stronger incentive to call patients within the simulation as the time penalty for doing so is low. Furthermore, a significant component of the workload in HTM systems comes from dealing with missed measurements or validating alerts that are out of range (often termed technical alerts). However, this would only impact on clinical workload if the clinician had a combined technical and clinical triage role. Our experiment assumes separate technical and clinical triage roles and that the alerts presented to participants have already been validated and missing alerts dealt with. Importantly these limitations affects only the total time and not the comparisons between each arms, which would be unaffected.

In a live telehealth system the healthcare professional will review the alerts on a daily basis and will develop an understanding of the patients that alert most often and why they alert. We are unable to simulate the entire time history of monitoring for a patient due to the time constraints of running the experiment. However, it is unknown whether greater familiarity with a patient leads to less or more time spent assessing them or indeed whether it might lead to clinical complacency.

## Conclusions

In a simulated setting comparing different alert algorithms we found that more advanced trend algorithms reduced the time needed to review alerts. However, although the reviews improved the discrimination of alerts in patients who later deteriorated, the agreement on actions between clinicians was moderate and bio-impedance alerts were often disregarded. More advanced alert algorithms presented in an intuitive way together with continuous evaluation of actions in a telemonitoring program will likely result in time efficiencies and improved clinical decision making. Simulation studies such as this one can help in the design process and determine putative benefits of introducing new technologies.

## Additional files

Additional file 1: Appendix 1. Study size calculation. (DOC 37 kb)
Additional file 2: Appendix 2. Proportion of actions suggested, intra class correlation, fleiss kappa and its components when split by algorithm. (DOC 219 kb)
Additional file 3: Appendix 3. De-identified participant level data of review times, proportion of ratings and proportion of actions. (DOC 66 kb)

## Abbreviations

CUSUM: Cumulative sum control chart
GUI: Graphical user interface
HF: Heart failure
HTM: Home telemonitoring
MACD: Moving average convergence divergence
NYHA: New York Heart Association
RCT: Randomised controlled trial
RoT: Rule of thumb
TTI: Trans-thoracic impedance
