# Supplementary material for: Simulated case management of home telemonitoring to assess the impact of different alert algorithms on work-load and clinical decisions
Source: BMC Med Inform Decis Mak. 2017 Jan 17;17:11. doi: 10.1186/s12911-016-0398-9 (PMC5240411; doi:10.1186/s12911-016-0398-9)
Supplement: Additional file 1: Appendix 1. — Study size calculation. (DOC 37 kb) [file 12911_2016_398_MOESM1_ESM.doc]

## Statistical considerations: study size argument

It is assumed that the study participant will only spend time responding to and reviewing actual alerts and will essentially ignore those patients not generating an alert. Consequently, the amount of time used will depend on the amount of alerts generated by each simulated case-load (i.e. weight + standard algorithms; weight + advanced algorithms; bio-impedance + advanced algorithms)

Arm-A produces approximately twice the number of alerts as the other two algorithm arms. Let us assume that the distribution of the time spent assessing alerts in the advanced Arm-P is normal (Gaussian), with a mean µ_P_ and a standard deviation σ_P_. Since the amount of time spent assessing alerts in Arm-A can be assumed to be twice that of Arm-P, we can express the distribution of time spent assessing alerts in Arm-A as the sum of two normal distributions;

N(*µ_A_*, *σ_A_^2^*) = N(*µ_P_*, *σ_P_^2^*) + N(*µ_P_*, *σ^2^_P_*)

Adding the two distributions together results in;

N(*µ_A_*, *σ_A_^2^*) = N(*µ_P_+ µ_P_*, *σ_P_^2^+σ_P_^2^*)

And therefore;

*µ_A_ = 2.µ_P_*

*σ_A_= √ (2. σ_P_^2^)*

If we assume a standard deviation of half the mean then for an alpha level of 0.05 and a power of at least 90%, 8 participants should be sufficient.
