# Supplementary material for: Simulated case management of home telemonitoring to assess the impact of different alert algorithms on work-load and clinical decisions
Source: BMC Med Inform Decis Mak. 2017 Jan 17;17:11. doi: 10.1186/s12911-016-0398-9 (PMC5240411; doi:10.1186/s12911-016-0398-9)
Supplement: Additional file 2: Appendix 2. — Proportion of actions suggested, intra class correlation, fleiss kappa and its components when split by algorithm. (DOC 219 kb) [file 12911_2016_398_MOESM2_ESM.doc]

## Detailed analysis of each algorithm


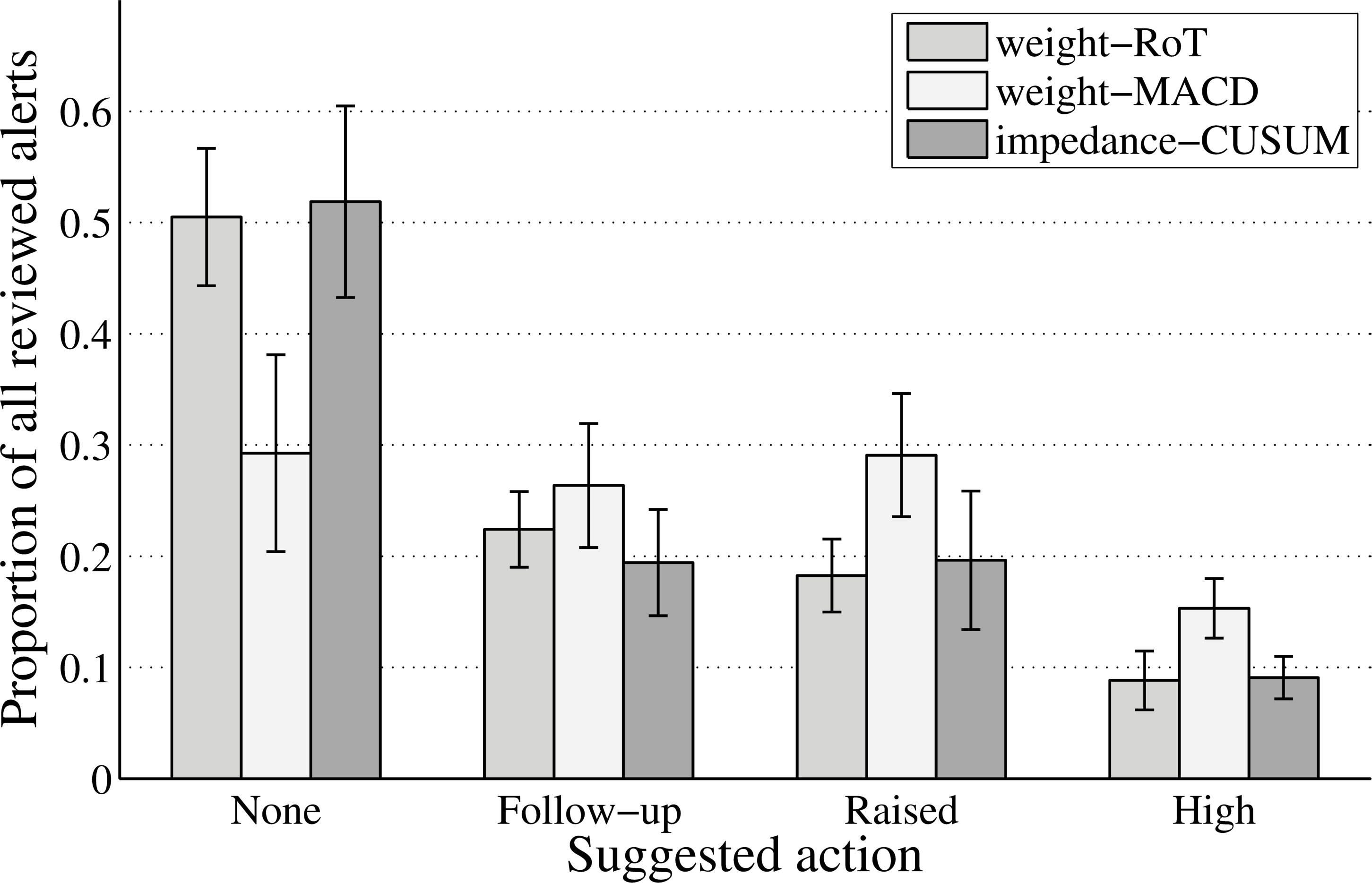


Figure 1: The mean and standard error of the proportion of the different actions suggested for the reviewed cases by the clinicians for each algorithm.

|  |  |  | **Fleiss κ component** | | | |
| --- | --- | --- | --- | --- | --- | --- |
|  | **ICC** | **Fleiss κ** | No Action | Follow-up | Raised concern | High concern |
| ***Weight- RoT*** | .462 (.413 − .514) | .155 (.152 – 0.158) | 0.209 | 0.076 | 0.157 | 0.223 |
| ***weight-MACD*** | .375 (.290 − .465) | .119 (.110 – .128) | 0.149 | 0.057 | 0.101 | 0.239 |
| ***impedance-CUSUM*** | .545 (.440 − .648) | .252 (.240 – .264) | 0.447 | 0.032 | 0.240 | 0.325 |

Table 1: Intra Class Correlation (C-1), Fleiss kappa and its components for the actions suggested by the participants for each algorithm together with 95% confidence intervals. The Fleiss kappa and ICC computation excluded cases in which not all clinicians provided a rating (total: *weight-RoT*, 281 cases with 8 raters; *weight-MACD*, 147 cases with 4 raters; and *impedance-CUSUM*, 85 cases with 4 raters).
