# Supplementary material for: Simulated case management of home telemonitoring to assess the impact of different alert algorithms on work-load and clinical decisions
Source: BMC Med Inform Decis Mak. 2017 Jan 17;17:11. doi: 10.1186/s12911-016-0398-9 (PMC5240411; doi:10.1186/s12911-016-0398-9)
Supplement: Additional file 3: Appendix 3. — De-identified participant level data of review times, proportion of ratings and proportion of actions. (DOC 66 kb) [file 12911_2016_398_MOESM3_ESM.doc]

## Raw data on review time, rating proportion, and action proportion.

| **User** | **Arm** | **Time (min)** | **Proportion of alert ratings (%)** | | | | | **Proportion of alert actions (%)** | | | |
| --- | --- | --- | --- | --- | --- | --- | --- | --- | --- | --- | --- |
|  |  |  | 1 | 2 | 3 | 4 | 5 | None | Follow-up | Raised | High |
| **1** | A | 66.8 | 0.38 | 23.40 | 49.81 | 21.51 | 4.91 | 21.07 | 41.47 | 32.44 | 5.02 |
| **2** | A | 112.2 | 0.00 | 56.40 | 29.76 | 9.34 | 4.50 | 61.20 | 23.75 | 10.70 | 4.35 |
| **3** | A | 133.6 | 0.76 | 65.91 | 28.41 | 4.55 | 0.38 | 64.55 | 10.03 | 13.04 | 12.37 |
| **4** | A | 72.1 | 0.00 | 17.34 | 48.34 | 29.52 | 4.80 | 72.95 | 12.46 | 11.74 | 2.85 |
| **5** | A | 81.4 | 4.27 | 32.48 | 17.95 | 37.61 | 7.69 | 45.82 | 23.08 | 27.42 | 3.68 |
| **6** | A | 201.5 | 0.34 | 17.41 | 79.86 | 2.39 | 0.00 | 54.52 | 27.09 | 6.35 | 12.04 |
| **7** | A | 267.4 | 21.4 | 29.77 | 28.09 | 15.72 | 5.02 | 53.51 | 22.07 | 19.06 | 5.35 |
| **8** | A | 257.0 | 1.38 | 30.80 | 49.83 | 17.65 | 0.35 | 30.33 | 19.33 | 25.33 | 25.00 |
| **9** | P(M) | 56.7 | 0.78 | 1.55 | 13.18 | 30.23 | 54.26 | 19.73 | 42.18 | 25.85 | 12.24 |
| **10** | P(M) | 42.1 | 8.76 | 40.88 | 14.60 | 25.55 | 10.22 | 55.78 | 16.33 | 16.33 | 11.56 |
| **11** | P(M) | 174.3 | 0.75 | 4.51 | 40.60 | 34.59 | 19.55 | 21.09 | 21.77 | 42.86 | 14.29 |
| **12** | P(M) | 57.0 | 1.40 | 12.59 | 23.78 | 42.66 | 19.58 | 20.41 | 25.17 | 31.29 | 23.13 |
| **13** | P(C) | 59.0 | 17.56 | 15.27 | 19.08 | 36.64 | 11.45 | 29.85 | 29.85 | 36.57 | 3.73 |
| **14** | P(C) | 84.3 | 40.35 | 18.42 | 20.18 | 14.91 | 6.14 | 68.66 | 14.18 | 6.72 | 10.45 |
| **15** | P(C) | 71.9 | 38.06 | 12.69 | 8.96 | 20.15 | 20.15 | 61.94 | 8.96 | 16.42 | 12.69 |
| **16** | P(C) | 92.5 | 41.46 | 6.10 | 10.98 | 34.15 | 7.32 | 47.06 | 24.71 | 18.82 | 9.41 |

Table 1: Participant level data on total review time (with reviews lasting longer than 5 minutes replaced with 5 minutes), the proportion of ratings given to the alert and the proportion of actions taken together with arm allocation (with a further distinction made for the algorithms in the advanced arm P: M, *weight-MACD*; C, *impedance-CUSUM*).
